# Strategic Task and Break Timing to Reduce Ultraviolet Radiation Exposure in Outdoor Workers

**DOI:** 10.3389/fpubh.2020.00354

**Published:** 2020-08-04

**Authors:** Cheryl E. Peters, Thomas Tenkate, Emily Heer, Rachel O'Reilly, Sunil Kalia, Mieke W. Koehoorn

**Affiliations:** ^1^Cancer Epidemiology and Prevention Research, Alberta Health Services, Calgary, AB, Canada; ^2^Cumming School of Medicine, University of Calgary, Calgary, AB, Canada; ^3^School of Occupational and Public Health, Ryerson University, Toronto, ON, Canada; ^4^Department of Dermatology and Skin Science, University of British Columbia, Vancouver, BC, Canada; ^5^School of Population and Public Health, University of British Columbia, Vancouver, BC, Canada

**Keywords:** ultraviolet radiation (UV), outdoor workers, exposure assessment (EA), solar UV, occupational cancer

## Abstract

**Objectives:** Public health messaging about sun avoidance strategies is often not practical for outdoor workers. The objective of this study was to use personal monitoring data to determine when peak UVR exposure occurs for outdoor workers, estimate how much UVR could be reduced by altering the timing of shady tasks or breaks during peak exposure times, and descriptively compare these to peak periods of ambient UVR. Ultimately, we aim to provide evidence-based sun avoidance recommendations for outdoor workers in British Columbia, Canada.

**Methods:** UVR exposure data [standard erythemal dose (SED)] were collected during the 2013 summer months in Vancouver, using personal electronic dosimeters that sampled once per minute for an average of 4.4 working days (range: 1–7 days). Mixed-effect models were used to estimate the 60-, 30-, and 15-min time intervals at which maximum exposure occurred for the months of July and August. Using these time intervals, UVR exposure during peak periods was summarized as SED and as a percentage of the total daily exposure. Ambient UVR was also collected using data from the nearest Brewer spectrophotometer station and parallel analyses were conducted.

**Results:** There were 73 workers and 321 participant-days available for analysis. Models indicated that periods of maximum exposure for 15-, 30-, and 60-min intervals began at 12:28, 12:17 pm, and 11:52 am, respectively, for sunny days in July. These periods were similar in August. The median exposure during these time periods and the potential for reducing UVR was 0.03 SED (2.8% potential daily exposure reduction), 0.09 SED (7.1%), and 0.18 SED (15.9%), respectively. However, there was a large range in exposure estimates as some workers experienced up to 84.8% of their exposure in the peak 60-min interval.

**Conclusion:** Skin cancer prevention messaging does not include practical messages for outdoor workers and providing times of peak UVR help to identify times when the greatest reductions in exposure can occur. Prevention measures including shady breaks, increased sun protection, and task reorganization during these peak times are recommended during these peak times to reduce UVR exposure among those at highest risk.

## Introduction

Outdoor workers are exposed to high levels of solar ultraviolet radiation (UVR) during the workday that leaves them at increased risk of developing skin cancer (1). This pattern of long-term exposure to solar UVR consistent with outdoor occupations is associated with an increased risk of non-melanoma skin cancers (NMSCs) (2, 3). Skin cancer is the most common cancer worldwide, and comprises almost 40% of incident cancers in Canada (4). Of these, almost all are NMSCs, and are largely preventable with effective sun protection (5). Recent estimates found that over 6% of NMSC cases in Canada were attributable to sun exposure at work, with the majority of these cases occurring among those in the agricultural or construction industries (6). Similar results have been found in other countries, suggesting that greater efforts need to be placed on providing resources for sun protection in these occupational settings (7, 8).

Prevention of excess UVR exposure has been the focus of some intervention studies among outdoor workers (9, 10). These interventions include the provision of UVR-protective clothing, sunscreen, and shade breaks at times of high UVR intensity (11, 12). However, the effectiveness of any of these interventions is inextricably linked to workplace culture and practicality (13). For instance, long-sleeved clothing and wide-brimmed hats may be uncomfortable or unsafe in some work environments (14). While shade-seeking can be an effective strategy for avoiding high UVR exposure and may be enforceable in some situations, it is not practical to recommend avoiding outdoor work during all peak UVR hours. The hours of highest UV intensity vary by region, but Environment Canada suggests avoiding outdoor activities between 11 am and 3 pm (15). This wide range is not a useful guide for most outdoor workers, particularly those in construction. Instead, it may be more beneficial for employers to be aware of the time of highest risk during the workday and to encourage breaks at these times.

The main purpose of this study was to provide evidence-based recommendations for sun avoidance for outdoor workers in British Columbia. Specifically, personal monitoring devices were used to determine when peak UVR exposure occurs for these workers, to estimate how much UVR could be reduced by altering the timing of shade tasks or breaks during peak exposure times, and to descriptively compare these periods to peak periods of ambient UVR in the same timeframe.

## Methods

This study was part of the Outdoor Workers Project based in Vancouver, British Columbia (49.28°N, 123.12°W). Data collection took place between July and early September of 2013 and included objective measures of solar UVR exposure; questionnaires were also collected with demographic information, skin cancer risk factors, job characteristics, and sun protection behaviors. Complete details of the project and the recruitment process can be found in previous publications (16, 17).

### UVR Measures

Personal electronic dosimeters (Mark II) were used to capture solar UVR exposure among the outdoor workers in construction and non-construction (mainly horticultural) sectors. Workers wore dosimeters either on a wrist band, pinned to the lapel, or on a hardhat, from 8:00 am to 5:00 pm each day (the difference in dose by body site was not statistically significant, and 85% of workers selected the lapel placement). These datalogging wireless devices take a measurement once per minute that can be translated to a UV Index measure and the standard erythemal dose (SED) for each day. Participants wore the dosimeters for an average of 4.4 days (minimum 1 day, maximum 7 days). The datalogging capability of this device allowed for time-stamped exposure measurements for each of the participants.

The SED is a measure of radiant exposure, and 1 SED is equivalent to 100 Joules per meter squared (18). Conversion from each measurement to a UV index measure and SED is described in a previous publication (16). Briefly, the dosimeters produce an analog output that is proportional to the UV radiation received on their face, and each dosimeter has a calibration curve relating the analog values to the UV index. The UV index per minute from the dosimeter is converted into an SED per minute using a standard conversion equation that takes solar irradiance into account, and is aggregated into a daily SED value by summing across the workday.

Data from the Brewer spectrophotometer station in Richmond was used to measure ambient UVR that is recorded every 10–20 min during daylight hours (19). Using on-site dosimeters to measure ambient UVR was not feasible due to the limited number of dosimeters available, and that researchers could not be on site to ensure safety of the equipment at multiple sites.

### Statistical Methods

Total SED for each participant-day were grouped based on reported number of hours worked outdoors (taking integer values from 1 to 6), and participant-days at or below the 5th percentile were excluded, based on the assumption that the dosimeter may not have been worn as intended. For Brewer spectrophotometer data, minute-level data was estimated by carrying forward the last measurement taken until a subsequent measurement was taken.

Times of maximum UVR exposure and ambient UVR were modeled by aggregating minute level UVR data within 15-, 30-, and 60-min periods. These time intervals were selected because they were deemed to be amenable to prevention strategies. For instance, it seems feasible to alter the scheduling of breaks that are 15–60 min in length. Quadratic mixed effect models were used to predict UVR based on the fixed effects time of day, Environment Canada's weather forecast characteristics (sun, cloud, or mixed), and the month (July, or August, with the two participant days obtained in early September added to August measures). The squared effect of time of day, as well as the interaction of this with month and with weather forecast, and associated lower order interaction terms were included as fixed effects within the models. We selected a quadratic model for two reasons; firstly, the relationship between time and exposure visually appeared to be quadratic in form, and secondly, using a quadratic model provided the advantage of being able to calculate the time at which maximum dose occurs by taking the derivative of the estimated model. The model took the following form:

$$\begin{array}{l} \text{ln}\left(\hat{\text{S}}\text{ED}\right)={\text{β}}_{0}+{\text{β}}_{1}\text{time}+{\text{β}}_{2}\text{tim}{\text{e}}^{2}+{\text{β}}_{3}\text{Aug}+{\text{β}}_{4}\text{cloud}\\ +{\text{β}}_{5}\text{mixed}+{\text{β}}_{6}\text{time}*\text{Aug}+{\text{β}}_{7}\text{time}2*\text{Aug}+{\text{β}}_{8}\text{time}*\text{cloud}\\ +{\text{β}}_{9}\text{time}2*\text{cloud}+{\text{β}}_{10}\text{time}*\text{mixed}+{\text{β}}_{11}\text{time}2*\text{mixed} \end{array}$$

Where:

- Time is a continuous covariate, where the series of values used depends on whether 15, 30, or 60 min intervals are used
- Month takes the values of July and August, where July is the reference level
- Weather forecast takes values “sun,” “cloud,” and “mixed,” where sun is the reference level

The natural logarithm of UVR (SED) was used to model the personal dosimeter measures, and the random effects in the model were participant identification number (ID), calendar date, and the interaction of participant ID and calendar date. The natural logarithm of UVR (SED) was not log-transformed when modeling the Brewer measures, and only a random effect for calendar date was included in this model. This approach provides a model-based estimation of the time at which periods of maximum worker exposure and ambient UVR occur.

Additionally, the absolute and relative proportions of daily UVR exposure were calculated for the peak-exposure periods predicted by each of the 15-, 30-, and 60-min participant dosimeter models. This yields an estimate of the amount and proportion of UVR that could be avoided by using a shade break at that time.

Analyses were conducted in SAS Enterprise Guide version 7.13 (20), StataSE 15 (21), and R Studio Version 1.1.447 (22). The study protocol was approved by the University of British Columbia's Behavioral and Research Ethics Board (certificate H11-01272).

## Results

In total, there were 73 workers and 321 participant-days included in this analysis. The majority of participants were young males working in marine and land-based construction (Table 1).

**Table 1:** Descriptive summary of study sample for UVR exposures in Vancouver, July—September 2013.

**Measure**	**Unique participants**	**Unique participant-days:**
	**or dates: n (%)**	**n (%)**
Participants (n)	73	321
Dates (n)	40	
Industry^a^		
Horticultural/non-construction	14 (19.2%)	54 (16.8%)
Land-based construction	29 (39.7%)	127 (39.6%)
Marine construction	30 (41.1%)	140 (43.6%)
Forecast^a^		
Sun	25 (62.5%)	250 (77.9%)
Mixed	8 (20.0%)	58 (19.1%)
Cloud	7 (17.5%)	13 (4.0%)
Month^a^		
July	15 (37.5%)	160 (49.8%)
August	23 (57.5%)	159 (49.5%)
September	2 (5.0%)	2 (0.6%)

a *Values for industry represent participants, values for forecast and month represent dates*.

### Dosimeter Measurements

Models for 60-min exposure periods predicted the hour of highest worker exposure beginning between 11:52 am and 12:19 pm, depending on month and weather forecast. All but two of the predicted peak times for 15- and 30-min models fell within the period predicted by the longer duration model. In general, models for sunny weather predicted earlier peak exposure periods, followed by cloudy and mixed-weather forecasts (Table 2). When considering the 60-min time interval, sunny days in July had the earliest estimated peak exposure period, beginning at 11:52 am. This result indicates that the model estimates highest exposure occurring between the times 11:52 am and 12:51 pm. The latest start time for a peak interval was for mixed-weather days in both July and August at 12:52 pm using the 15-min interval model.

**Table 2:** Participant dosimeter model for predicted times for periods of maximum exposure.

	**Time at start of period of maximum exposure^b^**	**95% confidence interval for time at start of period of maximum exposure**
15-min period model		
July—Sun	12:28 pm	12:20 pm−12:35 pm
July—Cloud^a^	12:30 pm	11:54 am−1:06 pm
July—Mixed^a^	12:52 pm	12:30 pm−1:15 pm
August—Sun	12:24 pm	12:15 pm−12:34 pm
August—Cloud	12:27 pm	11:50 am−1:05 pm
August—Mixed	12:52 pm	12:34 pm−1:11 pm
30-min period model		
July—Sun	12:17 pm	12:07 pm−12:26 pm
July—Cloud^a^	12:33 pm	11:47 am−1:19 pm
July—Mixed^a^	12:41 pm	12:13 pm−1:09 pm
August—Sun	12:11 pm	11:59 am−12:23 pm
August—Cloud	12:29 pm	11:42 am−1:17 pm
August—Mixed	12:37 pm	12:15 pm−1:00 pm
60-min period model		
July—Sun	11:52 am	11:43 am−12:02 pm
July—Cloud^a^	11:57 am	11:10 am−12:45 pm
July—Mixed^a^	12:10 pm	11:46 am−12:34 pm
August—Sun	11:54 am	11:38 am−12:10 pm
August—Cloud	12:01 pm	10:56 am−1:05 pm
August—Mixed	12:19 pm	11:53 am−12:45 pm

a *The study did not sample any days that had a forecast of cloud or mixed in July. Thus, these estimates are extrapolated based on the combination of the differences in timing between July and August, and between sun, cloud, and mixed forecasts within August*.

b *The estimated times represent the start of the peak expsure period (i.e., the estimated peak hour exposure period for sunny days in July is 11:52 am−12:51 pm and the 15-min exposure period for mixed days in August is 12:52–1:06 pm)*.

Table 3 presents the absolute and relative amounts of UVR exposure participants receive during the peak periods identified by participant dosimeter models. The highest absolute and relative median exposure for each time interval occurred during sunny days in July, while the smallest occurred on cloudy days in August (Table 3).

**Table 3:** Absolute and relative UVR exposure during peak periods identified by participant dosimeter models.

		**Peak 60-min period (*****n***^**a**^ **= 321)**		**Peak 30-min period (*****n***^**a**^ **= 321)**		**Peak 15-min period (*****n***^**a**^ **= 321)**	
**Measure**	**Total daily exposure(SED)^**b**^**	**Exposure (SED)^**c**^**	**Percent of daily exposure^**d**^**	**Exposure (SED)^**d**^**	**Percent of daily exposure^**d**^**	**Exposure (SED)^**d**^**	**Percent of daily exposure^**d**^**
**ALL DATES**							
Mean	2.41	0.48	17.02	0.24	8.38	0.12	4.16
Maximum	19.14	3.50	84.76	3.10	81.23	2.13	55.81
Upper quartile	3.27	0.66	23.68	0.30	11.62	0.15	6.13
Median	1.34	0.18	15.94	0.09	7.08	0.03	2.79
Lower quartile	0.57	0.05	7.56	0.01	1.35	0.00	0.00
		**Peak 60-min period (*****n***^**a**^ **= 160)**		**Peak 30-min period (*****n***^**a**^ **= 160)**		**Peak 15-min period (*****n***^**a**^ **= 160)**	
**Measure**	**Total daily UVR (SED)**^**b**^	**UVR Exposure (SED)**^**d**^	**Percent of daily exposure**^**d**^	**UVR Exposure (SED)**^**d**^	**Percent of daily exposure**^**d**^	**Exposure (SED)**^**d**^	**Percent of daily exposure**^**d**^
**JULY SUN**							
Mean	3.10	0.66	19.29	0.33	9.44	0.17	4.77
Maximum	19.14	3.50	84.76	3.10	81.23	2.13	55.81
Upper quartile	3.95	0.90	24.72	0.47	12.11	0.24	6.42
Median	2.01	0.36	18.12	0.16	8.55	0.07	4.01
Lower quartile	0.83	0.10	11.79	0.04	4.02	0.02	1.35
		**Peak 60-min period (*****n***^**a**^ **= 90)**		**Peak 30-min period (*****n***^**a**^ **= 88)**		**Peak 15-min period (*****n***^**a**^ **= 88)**	
**Measure**	**Total daily UVR (SED)**^**b**^	**Exposure (SED)**^**d**^	**Percent of daily exposure**^**d**^	**Exposure (SED)**^**d**^	**Percent of daily exposure**^**d**^	**Exposure (SED)**^**d**^	**Percent of daily exposure**^**d**^
**AUGUST SUN**							
Mean	2.29	0.40	15.79	0.19	7.39	0.09	3.43
Maximum	9.66	2.35	54.66	1.13	33.12	0.50	26.23
Upper quartile	3.72	0.49	22.44	0.21	11.46	0.12	5.47
Median	1.41	0.17	13.06	0.08	6.16	0.02	2.29
Lower quartile	0.57	0.03	3.50	0.00	0.2	0.00	0.00
		**Peak 60-min period (*****n***^**a**^ **= 13)**		**Peak 30-min period (*****n***^**a**^ **= 13)**		**Peak 15-min period (*****n***^**a**^ **= 13)**	
**Measure**	**Total daily UVR (SED)**^**b**^	**Exposure (SED)**^**d**^	**Percent of daily exposure**^**d**^	**Exposure (SED)**^**d**^	**Percent of daily exposure**^**d**^	**Exposure (SED)**^**d**^	**Percent of daily exposure**^**d**^
**AUGUST CLOUD**							
Mean	0.26	0.04	18.98	0.03	10.31	0.02	6.15
Maximum	1.14	0.17	60.69	0.13	49.29	0.12	30.14
Upper quartile	0.27	0.07	31.77	0.03	10.01	0.01	5.09
Median	0.14	0.02	14.07	0.00	3.68	0.00	2.44
Lower quartile	0.11	0.00	0.00	0.00	0.00	0.00	0.00
		**Peak 60-min period (*****n***^**a**^ **= 58)**		**Peak 30-min period (*****n***^**a**^ **= 58)**		**Peak 15-min period (*****n***^**a**^ **= 58)**	
**Measure**	**Total daily UVR (SED)**^**b**^	**Exposure (SED)**^**d**^	**Percent of daily exposure**^**d**^	**Expsure (SED)**^**d**^	**Percent of daily exposure**^**d**^	**Exposure (SED)**^**d**^	**Percent of daily exposure**^**d**^
**AUGUST MIXED**							
Mean	1.19	0.20	12.22	0.10	6.50	0.05	3.14
Maximum	6.71	1.53	52.93	0.75	34.76	0.43	21.84
Upper quartile	1.51	0.18	18.45	0.10	9.26	0.04	3.74
Median	0.58	0.07	10.84	0.02	3.91	0.01	1.61
Lower quartile	0.29	0.00	0.00	0.00	0.00	0.00	0.00

*July cloud and July mixed were not included in this summary because no days with these forecasts were sampled*.

a *n refers to the number of participant-days considered within the rows describing a combination of month and weather forecast*.

b *Total daily exposure was calculated as the sum of SED/minute across 8:00 am−4:59 pm, weighted by number of non-missing values*.

c *Absolute exposure during this time period was calculated as the sum of SED/minute across all minutes within time period, weighted according to number of non-missing values. For example, the half hour period for sunny days in July includes the minutes 12:17–12:46 pm*.

d *Percent of daily exposure was calculated as: 100% × exposure during peak period / total daily exposure*.

### Brewer Spectrophotometer

Data on ambient UVR was gathered from the Brewer spectrophotometer station to assess periods of peak ambient UVR and compare to those estimated using participant dosimeters (Table 4). Overall, the times of maximum ambient UVR were assessed to be later compared to the personal dosimeter measurements: the earliest start time for an estimate of peak UVR was predicted to be 12:51 pm (60-min model, August, sun) and the latest estimate began at 1:34 pm (15-min model, July, cloud).

**Table 4:** Predicted start times for periods of maximum ambient UVR using Brewer spectrophotometer data.

	**Time at start of period**	**95% confidence interval**
	**of maximum exposure**	
15-min period model		
July—Sun	1:13pm	1:09–1:16 pm
July—Cloud^a^	1:34pm	1:19–1:49 pm
July—Mixed^a^	1:27pm	1:15–1:39 pm
August—Sun	1:08pm	1:03–1:12 pm
August—Cloud	1:30pm	1:17–1:47 pm
August—Mixed	1:21pm	1:12–1:31 pm
30-min period model		
July—Sun	1:07pm	1:02–1:11 pm
July—Cloud^a^	1:29pm	1:10–1:48 pm
July—Mixed^a^	1:21pm	1:06–1:36 pm
August—Sun	1:01pm	12:56–1:06 pm
August—Cloud	1:24pm	1:08–1:40 pm
August—Mixed	1:14pm	1:02–1:26 pm
60-min period model		
July—Sun	12:57*pm*	12:52–1:02 pm
July—Cloud^a^	1:22pm	12:58–1:45 pm
July—Mixed^a^	1:11pm	12:53–1:30 pm
August—Sun	12:51pm	12:44–12:57 pm
August—Cloud	1:14pm	12:55–1:34 pm
August—Mixed	1:03pm	12:49–1:17 pm

*The estimated times represent the start of the peak ambient UVR period (i.e., the estimated peak hour exposure period for sunny days in July is 12:57–1:56 pm and the 15-min exposure period for cloudy days in August is 1:30–1:59 pm)*.

a *We did not sample any days that had a forecast of cloud or mixed in July. Thus, these estimates are extrapolated based on the combination of the differences in timing between July and August, and between sun, cloud, and mixed forecasts within August*.

**Figure 1:**
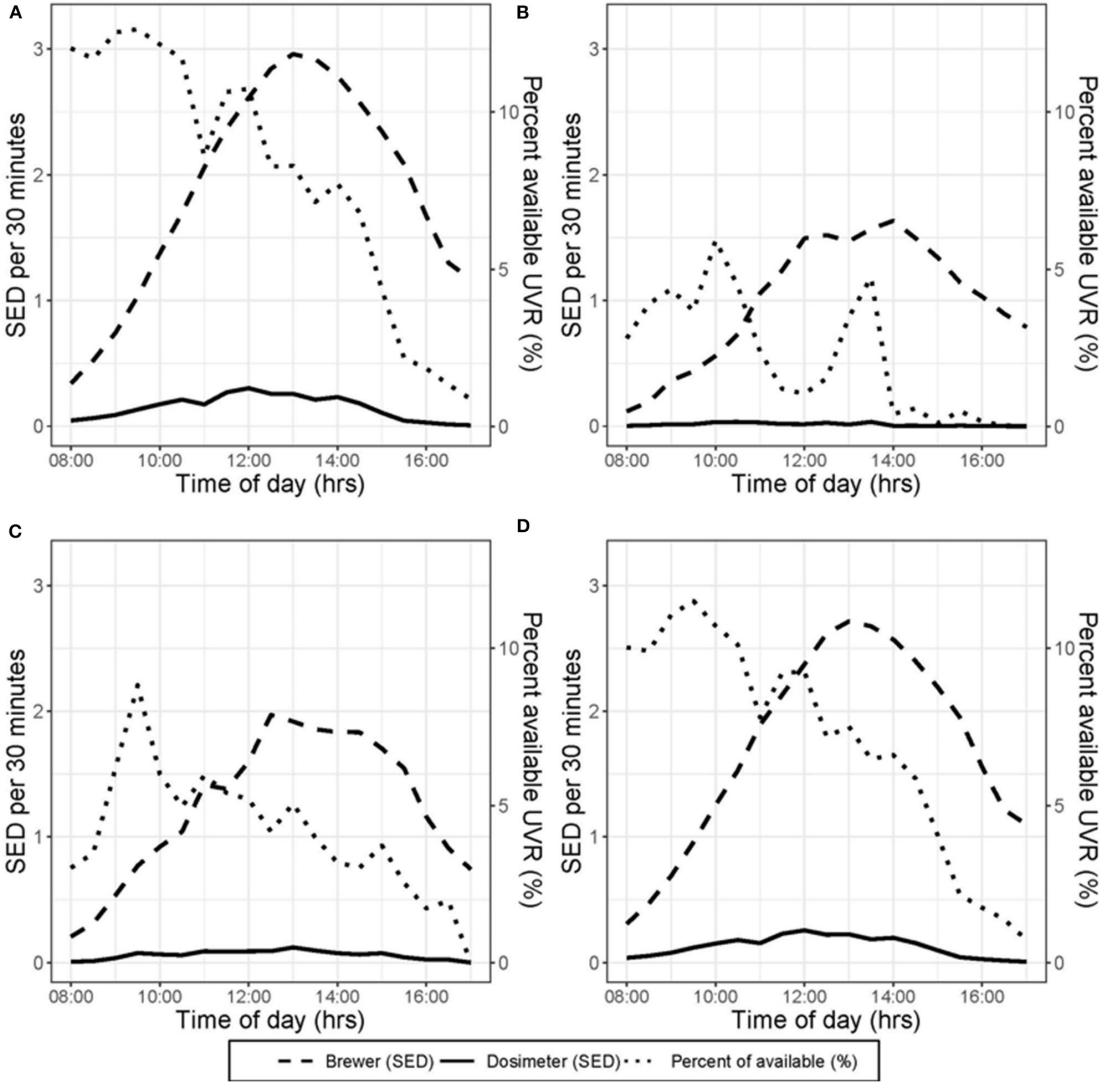
Depiction of the mean UVR measured by participant dosimeters, mean UVR measured by the Brewer spectrophotometer, and mean participant dosimeter UVR as a percentage of Brewer spectrophotometer UVR. Data are aggregated in 30-min periods. **(A–C)** Show days with forecast characteristics of sun, cloud and mixed, respectively. **(D)** Shows all days included in analysis.

Figures 1A–D shows the arithmetic means of ambient and personal UVR, and dosimeter proportion of the ambient UVR exposure by time of day. As described previously, peak ambient UVR on sunny days occurred in early to mid-afternoon, whereas the greatest amount of UVR measured by participant dosimeters was obtained during the late morning and peaked at noon (Figure 1A). This pattern is similar for all weather forecasts (cloud, or mixed, and all weather forecasts combined) (Figures 1B–D).

## Discussion

The results from this study provide evidence for policy makers or employers on how to protect employees from the highest levels of UVR exposure within a Canadian context. Specifically, we identified times of peak exposure for a subset of outdoor workers in Vancouver in July and August using UVR measures obtained from both personal dosimeters and the Brewer spectrophotometer station in the Vancouver region. Measurements using personal dosimeters gave peak exposure “start” times of between 11:52 am and 12:52 pm, depending on month and weather type (sun, cloud, or mixed), whereas measurements from the spectrophotometer station gave peak “start” times ~1 h later (between 12:51 and 1:34 pm). These results suggest that if workers were to avoid sun exposure during the times identified with dosimeter data, and not compensate with exposure at other times of day, they could avoid between 2.8 and 15.9% of their daily UVR exposure (based on median SED exposure for “all dates”), depending on the amount of time using avoidance strategies (15, 30, or 60 min). It is important to reiterate that we are not recommending workers take a break in the shade in these high-exposure times but then spend time outdoors during the peak times estimated by the Brewer spectrophotometer. Instead, personal data from the workers coupled with ambient measures from the Brewer suggest that sun avoidance should be encouraged as much as possible between 11:30 AM and 1:30 PM at latitudes similar to Vancouver, Canada.

The discrepancy between peak UVR timing as measured by personal dosimeters and the Brewer spectrophotometer provides information about the behavior of this study sample. Specifically, either it is a coincidence based on the types of tasks typically performed near midday, or workers may already be avoiding the sun during the peak ambient exposure times resulting in lower personal peak measurements compared to the ambient measurements for the same time. We did not collect information from the workers regarding their break timing, so this idea remains speculative in nature. This avoidance could be for reasons other than UVR protection; in previous work we found only a small proportion of workers use shade-seeking as a method of sun safety (17). In this case, workers may be beginning the lunch break during the period between 12:51 and 1:34, where we noted the highest ambient UVR measures. This can be interpreted as an unintentional sun protection strategy. However, the ambient UVR is still high prior to and following the lunch break, so prevention efforts remain necessary broadly across the midday period.

To our knowledge, this is the first study to model the ideal timing of sun avoidance behaviors among outdoor workers in Canada. A similar study from New Zealand also analyzed peak daily UVR exposure of construction workers using dosimeter and ambient measures (23). In this sample, the highest personal exposure occurred between 2:00 and 3:00 pm, while the highest ambient UVR peaked between 12:00 and 1:00 pm. As in our study, this indicated that workers were likely seeking shade during the time of greatest ambient UVR exposure, and then returning to work when ambient UVR was lower (23). A study from Australia in 1999 estimated human UVR exposure with horizontally mounted UV Biometer (24). Although this study did not directly measure worker exposure to UVR, the results showed that an indoor break between 11:30 am and 1:30 pm would reduce daily exposure by up to 20%. If this break were complemented by task reorganization between 11:00 am and 1:00 pm, the reduction in daily UVR exposure could reach 40% (24). These results and the recommendations for prevention are similar to those given by the present study and provide additional evidence for shady breaks at midday.

Previous studies on sun protection among outdoor workers have examined the role of sun avoidance or shade-seeking and the use of these strategies in outdoor occupations. Information on sun protection strategies was collected in this cohort for a previous analysis, and shade-seeking was found to be the least common form of sun protection behavior at work. Only 8% of employees reported “often” or “always” staying in the shade at work, while the most common behavior was wearing a shirt with long sleeves (82%) (17). In general, shade-seeking is an uncommon practice among outdoor workers and may reflect a lack of shade availability (25). However, there are reports of this practice being used in higher proportions among outdoor workers in other areas. In Florida, for example, 40% of workers from a variety of outdoor occupations reported seeking shade, but to avoid high heat, not necessarily to avoid UV exposure (26). This higher proportion could be explained by the fact that participants were asked about strategies used to avoid heat exposure rather than to avoid sun (UVR) exposure, or that the climate in Florida necessitates a greater number of shade breaks than in other locations. The first negative consequence of excess UVR exposure (sunburn) occurs hours after first exposure but heat is immediately apparent to workers, which may explain why shade seeking to protect against heat stress is reported more frequently by outdoor workers.

The results from this study show that reductions in UVR exposure can be achieved by strategically timing shady breaks. Overall, median exposure during peak hour was 0.18 SED (15.9% of daily exposure) but there is substantial variability, with the maximum 1-h exposure being almost 85% of that workers daily UV exposure. Therefore, shade seeking may be significant for those in the highest exposure categories. While it is useful to know the times of optimal sun avoidance, it is also important that workplaces provide the means and support to ensure that employees can seek shade during these times. As discussed previously, shade seeking at midday may not be practical in some situations (i.e., roadside construction), and may also be discouraged because of employer perceptions (i.e., lost productivity or financial constraints) (14). To confront these barriers, several studies have reported that it is important for workplaces to establish a culture that promotes prevention, including resources for protection, employee training, and policy or mandates (13, 27–29). Participants in a qualitative study on the barriers to and facilitators of sun safety interventions discussed the importance of senior-level engagement in workplace health promotion (30). Such a culture may be most effectively developed through implementing a workplace Sun Safety Program (31).

While this study is novel and provides insights into how to protect Canadian outdoor workers from dangerous levels of UVR, the results should be interpreted in the context of several limitations related to the study design and sample recruitment. The sample included in this analysis is fairly homogenous, and participants, and sampling dates were based on convenience sampling. Additionally, every day sampled in July had a forecast of sun, and sampled forecasts were not normalized against average weather in Vancouver, which may impact generalizability. While this means that the exposure estimates cannot be translated for forecasts without full sun, the results do reflect the highest possible exposure for July and is useful for mitigation efforts. Similarly, these results only apply to summer months and prevention efforts will likely differ with seasonality. Further, times of peak UVR exposure will differ by geographic region, so similar studies should be conducted in areas of different latitude and climate. Finally, we were unable to statistically compare the ambient UV data from the Brewer machine with the personal UV data from the dosimeters, due to the complexity of the correlation structure of the data. However, using measures of both participant exposure and ambient UVR provided a unique insight into worker behavior that we may not have discerned otherwise. Finally, we did not collect information from the workers on the timing of their breaks, nor whether they were strategically shifting tasks to times with lower ambient UVR on purpose.

Ultimately, this study provides evidence-based recommendations about skin cancer prevention for those who work in outdoor occupations. By modeling data from both personal and ambient UVR measures, we were able to highlight peak times of exposure during summer months for outdoor workers in Vancouver (between 11:30 AM and 1:30 PM). During these times, employees should seek shade or use additional sun protection methods. While these recommendations are directed toward employee practices in the construction industry [given that this was the largest industry represented in our study participants, and that UVR exposure varies widely by profession (32)], other employers should also support employee practices by providing shade options and task rotation or flexibility where possible during peak exposure periods, and by adopting a culture that encourages workers to reduce exposure to UVR as a known carcinogen. In the future, trials should be conducted with interventions that provide shade to workers, in order to provide evidence for shade-seeking during peak times.

## Data Availability Statement

The datasets presented in this article are not readily available because only aggregated data was deemed allowable to share beyond the research team by the ethics board. Requests to access the datasets should be directed to Cheryl E. Peters, cheryl.peters@ahs.ca.

## Ethics Statement

The studies involving human participants were reviewed and approved by University of British Columbia's Behavioural and Research Ethics Board, certificate H11-01272. The patients/participants provided their written informed consent to participate in this study.

## Author Contributions

CP designed the field study with the input of MK and SK, collected the UV dosimetry data, contributed to the idea for the present analysis, co-wrote the manuscript, and revised versions. TT also contributed to the idea for the present analysis, provided guidance on the methods, and contributed to each revision of the manuscript. EH wrote a significant portion of the manuscript and including interpretation of the results. RO'R devised the method to calculate peak exposure times and ran the analyses to do so and provided key input on the manuscript, particularly in the methods section. SK provided input on the field study, including study design, recruitment, contributed to manuscript revisions and including interpretation of the results. MK also contributed to the idea for the present analysis, supported development of the field study protocol, provided key input to the analysis, and interpretation of the results (and revisions of the manuscript). All authors contributed to the article and approved the submitted version.

## Conflict of Interest

The authors declare that the research was conducted in the absence of any commercial or financial relationships that could be construed as a potential conflict of interest.
